# Comparative Effects of Cellulose- and Gelatin-Based Hemostatic Biomaterials on the Early Stage of Wound Healing—An In Vivo Study

**DOI:** 10.3390/jfb17020064

**Published:** 2026-01-27

**Authors:** Helena Hae In Ströthoff, Polina Shabes, Katharina Henrika Beckamp, Markus Udo Wagenhäuser, Wiebke Ibing, Julian-Dario Rembe, Hubert Schelzig, Waseem Garabet

**Affiliations:** Department of Vascular and Endovascular Surgery, University Hospital of Düsseldorf, Heinrich-Heine-University Düsseldorf, Moorenstraße 5, 40225 Düsseldorf, Germany; helena.stroethoff@web.de (H.H.I.S.); polina.shabes@med.uni-duesseldorf.de (P.S.); katharinahenrika.beckamp@med.uni-duesseldorf.de (K.H.B.); markus.wagenhaeuser@med.uni-duesseldorf.de (M.U.W.); wiebke.ibing@med.uni-duesseldorf.de (W.I.); julian-dario.rembe@med.uni-duesseldorf.de (J.-D.R.); hubert.schelzig@med.uni-duesseldorf.de (H.S.)

**Keywords:** hemostatic biomaterials, wound healing, inflammation, growth factors, oxidized cellulose, gelatin

## Abstract

Hemostatic biomaterials are widely used in surgical and trauma settings, yet their influence on early wound healing remains incompletely understood. This in vivo study investigated the effects of cellulose- and gelatin-based hemostatic biomaterials on early wound healing using a murine skin wound model. Oxidized non-regenerated cellulose (ONRC), oxidized regenerated cellulose (ORC), and a porcine gelatin-based matrix (GELA) were left in situ following standardized subcutaneous implantation and compared with sham-treated controls. Tissue responses were analyzed at postoperative days 3 and 7 using histology, immunohistochemistry, and quantitative real-time polymerase chain reaction (qPCR). Cellulose-based materials persisted as eosinophilic remnants, whereas fibrous matrix structures and enhanced extracellular matrix deposition were observed in the GELA group. Immunohistochemical analysis revealed increased cluster of differentiation 68 (CD68)–positive macrophage presence in the ORC group at day 3 and in the GELA group at day 7, indicating biomaterial-dependent modulation of macrophage involvement during early wound healing. Expression of Kiel 67 (Ki-67), a marker of cellular proliferation, was significantly elevated in the epidermis of the GELA group at day 7, suggesting enhanced proliferative activity during the reparative phase. In contrast, no significant differences were detected in the expression of interleukin-6 (IL-6), tumor necrosis factor alpha (TNF-α), or cluster of differentiation 14 (CD14) between groups. Overall, none of the investigated biomaterials impaired early wound healing, while the gelatin-based material demonstrated features consistent with enhanced reparative cellular responses without excessive inflammation.

## 1. Introduction

Hemostatic biomaterials are routinely employed in a wide range of surgical procedures and trauma settings to control bleeding and are often intentionally left in situ to maintain hemostasis. Beyond their primary role in coagulation, these materials can significantly influence the wound healing process due to their diverse compositions and physicochemical properties, each of which presents distinct advantages and limitations [1,2].

Wound healing is a complex, highly coordinated process that progresses through overlapping phases: hemostasis, inflammation, proliferation (including granulation tissue formation and angiogenesis), and tissue remodelling [3,4]. Among these phases, inflammation is critical for initiating tissue repair. It begins immediately after injury and typically lasts 48–96 h. This phase is characterized by platelet aggregation, immune cell recruitment, and the release of proinflammatory cytokines such as tumour necrosis factor-alpha (TNF-α), interleukin-1 (IL-1), and interleukin-6 (IL-6) [5,6]. These cytokines play a pivotal role in attracting neutrophils and macrophages to the wound site. Neutrophils dominate the early response, contributing to pathogen clearance and reactive oxygen species (ROS) production [7,8]. Subsequently, macrophages assume a central role by clearing apoptotic cells and debris and by transitioning from a proinflammatory (M1) to a reparative (M2) phenotype. This phenotypic switch is essential for resolving inflammation and promoting tissue regeneration [5,6,7,9].

Collagen is another key component of wound healing, contributing across multiple stages. During hemostasis, it provides structural support and enhances clot formation [10]. Collagen also helps regulate inflammation by absorbing wound exudate and cytokines, thereby creating a conducive environment for immune cell activity [11]. During the proliferative phase, it promotes fibroblast activation, angiogenesis, and granulation tissue formation [12]. Furthermore, collagen facilitates epithelialization by supporting keratinocyte migration and proliferation, accelerating wound closure [13]. Conversely, dysregulated collagen deposition may impair healing or result in fibrosis [11,12].

Given their interactions with biological tissues, hemostatic biomaterials can substantially modulate the early wound environment, particularly during the inflammatory phase. For instance, gelatin sponges and chitosan-based hydrogels have been shown to mitigate excessive inflammation through the controlled release of anti-inflammatory molecules, thereby stabilizing the wound microenvironment and reducing tissue damage associated with prolonged inflammatory responses [14,15]. Among the most commonly used hemostatic agents in clinical practice are cellulose- and gelatin-based biomaterials, particularly in perioperative settings, where their effects on inflammation and healing remain under investigation [2].

While several in vitro studies have explored the effects of various hemostatic biomaterials on cellular behaviour and cytokine expression, their differential impact on wound healing—particularly on early inflammatory responses in vivo—remains inadequately characterized.

In this study, we aimed to investigate the early inflammatory effects of three clinically relevant hemostatic biomaterials in an animal model of wound healing. Specifically, we examined the gelatin-based material GELITA^®^ TUFT-IT (GELA), oxidized regenerated cellulose Tabotamp^®^ (ORC), and oxidized non-regenerated cellulose Resorba CELL^®^ (ONRC). By analyzing local tissue responses at 3 and 7 days post-application, we focused on cellular infiltration and the expression of key proinflammatory cytokines, including TNF-α and IL-6, to better understand how these biomaterials influence the early phases of wound healing.

The novelty of this study lies in the direct in vivo comparison of oxidized regenerated cellulose, oxidized non-regenerated cellulose, and a gelatin-based hemostatic biomaterial under identical experimental conditions, with a specific focus on the early phase of wound healing. By combining histological, immunohistochemical, and molecular analyses at defined early time points, this study provides integrated insight into biomaterial-specific cellular responses that are not sufficiently addressed by existing in vitro or single-material studies.

Current challenges associated with hemostatic biomaterials include excessive or prolonged inflammation, impaired cellular proliferation, and delayed tissue remodelling. By systematically evaluating macrophage involvement, proliferative activity, and inflammatory marker expression in vivo, the present study contributes to a better understanding of how commonly used biomaterials interact with early wound healing processes, thereby supporting more informed biomaterial selection in clinical practice.

## 2. Methods

### 2.1. Investigated Hemostatic Biomaterials

Three different hemostatic biomaterials were investigated in this study (Table 1): two cellulose-based materials, oxidized non-regenerated cellulose (ONRC) and oxidized regenerated cellulose (ORC), and one porcine gelatin-based material (GELA). Cellulose is a plant-derived, biodegradable polysaccharide and a major structural component of the primary cell wall in green plants. It consists of glucopyranose units linked by β-glycosidic bonds. Regenerated cellulose forms organized fibres, whereas non-regenerated cellulose retains a disorganized structure prior to oxidation [16]. In contrast, gelatin is a hydrocolloid produced by partial acid hydrolysis of porcine collagen, which is subsequently aerated into a foam and dried to create a sponge-like material [17].

According to manufacturer specifications, all three agents are absorbable. ONRC and ORC are typically absorbed within 14 days, whereas GELA is expected to be absorbed within 30 days. For intraoperative application in this study, a 1 × 1 cm piece of each hemostatic biomaterial was used per mouse.

### 2.2. Experimental Animal Model

The animal model was selected based on both clinical relevance and practical considerations to ensure maximal translational value of the experimental findings to the clinical setting. A small animal model using mice (Mus musculus, C57BL/6J) was chosen for this study. To eliminate variability related to hormonal cycling, only fully grown male animals aged 8 to 16 weeks were included.

The animals were sourced from Janvier Labs (Le Genest-Saint-Isle, France) and the Central Institute for Animal Research and Scientific Animal Welfare (Zentrale Einrichtung für Tierforschung und wissenschaftliche Tierschutzaufgaben; ZETT, Düsseldorf, Germany). All procedures were reviewed and approved by the competent authority under reference number [81-02.04.2021.A354] and were conducted in accordance with applicable regulations and ethical guidelines for animal experimentation.

### 2.3. Experimental Design

The animals were divided into four groups: three intervention groups, each receiving one hemostatic biomaterial, and a sham group undergoing surgery without implantation of a biomaterial.

- Group I—Sham (control group): No implantation of a hemostatic biomaterial
- Group II—GELA: Porcine gelatin-based hemostatic biomaterial
- Group III—ONRC: Oxidized non-regenerated cellulose
- Group IV—ORC: Oxidized regenerated cellulose

**Figure 1:**
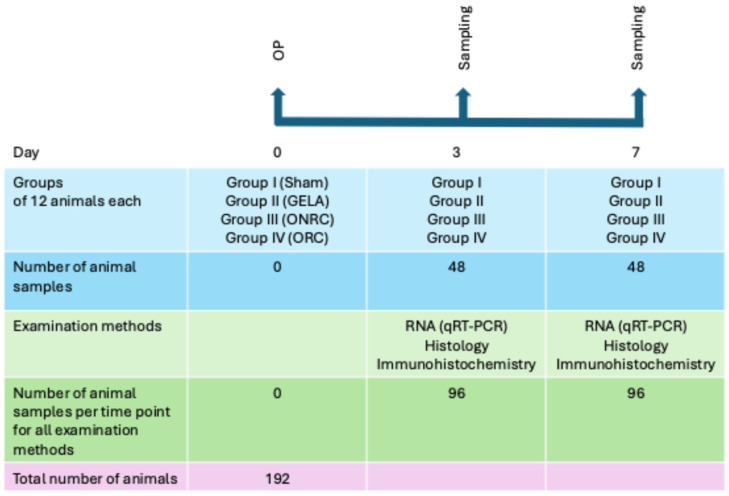
Study design. Overview of the study design and the division into 4 groups as well as the resulting number of mice and used methods.

As tissue processing differed depending on the analytical method, a separate group (n = 12) was required for each planned analysis. To assess outcomes during the early phases of wound healing, sample collection was performed on postoperative days 3 and 7, resulting in a total of 192 mice undergoing surgery (Figure 1).

Animals displaying visible signs of illness or injury prior to surgery were excluded. No retrospective exclusions were made unless predefined criteria were met (e.g., unrelated unexpected health decline). Variations in sample size resulted from layer-specific technical limitations.

### 2.4. Surgical Procedure and Monitoring

Following an acclimatization period of exactly 1 week, surgical procedures were performed. Prior to surgery, animals were weighed, clinically examined, and anesthetized using a weight-adjusted dose of ketamine/xylazine. Surgical preparation included shaving, disinfection with Octeniderm^®^ (Schülke & Mayr GmbH, Norderstedt, Germany), and sterile draping of the surgical field. During surgery, animals were positioned on a heating pad maintained at 37 °C. A 2 cm dorsal skin incision was made, and a 1 cm^2^ piece of hemostatic biomaterial was placed subcutaneously on the muscle fascia. The wound was closed using Prolene 3-0 sutures.

Postoperative analgesia was administered orally with metamizole (1.33 mg/mL) for the first two days following surgery. Animals were monitored daily during the first postoperative week for wound condition, behaviour, and general health using a standardized scoring system provided by the animal facility. Thereafter, assessments were conducted weekly. The experiment was terminated immediately if predefined humane endpoints were met, including ≥20% body weight loss, respiratory distress, self-isolation, paralysis, extensive wound infection, tail necrosis, or severe bleeding. Overall, this subcutaneous surgical model was considered low-stress, and anaesthesia was administered prior to all invasive procedures.

Wounds were photographed and tissue samples collected on postoperative days 3 and 7, which constituted the experimental endpoints. Prior to sample collection, animals were premedicated with metamizole and anesthetized using isoflurane. Cardiac blood was collected (maximum 1 mL), followed by euthanasia via cervical dislocation. Tissue samples measuring 2 × 2 cm—including cutis, subcutis, and underlying connective tissue—were excised from the wound area for further analysis. After excision, one quarter of each tissue sample was fixed in 4% paraformaldehyde (PFA; Rockland, ME, USA) and moulded in kerosene.

### 2.5. Histological and Immunohistochemical Analysis

Following fixation in 4% PFA, tissue samples were embedded and sectioned at a thickness of 4.5 µm. Histological evaluation was performed using hematoxylin–eosin (HE) and Movat Pentachrome (MP) staining, conducted according to standard protocols (MORPHISTO GmbH, Offenbach, Germany).

For immunohistochemical analysis, two primary antibodies were used: Ki-67 (Kiel 67; antibodies-online GmbH, Aachen, Germany), a marker of cellular proliferation, and CD68 (Invitrogen, Carlsbad, CA, USA, ThermoFisher, Waltham, MA, USA), a marker of macrophage activation. The Ki-67 proliferation index was calculated as the ratio of Ki-67–positive cells to the total number of cells within the tissue section.

The staining protocol was conducted over three days. On day 1, tissue sections were deparaffinized, subjected to antigen retrieval, and blocked with goat serum. Primary antibodies (Ki-67 and CD68) were applied and incubated overnight. On day 2, sections were washed and incubated with fluorescent secondary antibodies (Alexa Fluor 488 and 594), followed by nuclear counterstaining with DAPI. Slides were mounted using Mowiol mounting medium. On day 3, fluorescence microscopy was performed using specific detection channels: Ki-67 (red), CD68 (green), and DAPI (blue). Images were captured at 40× magnification.

Quantitative analysis of immunohistochemical staining was performed using the open-source software ImageJ version 1.49 (National Institutes of Health, Bethesda, MD, USA) [18]. TIFF-format microscopy images were analyzed to determine mean fluorescence intensity of CD68 and Ki-67 per cell. In addition, DAPI-stained nuclei were counted. For increased precision, epidermal and dermal layers were analyzed separately. Hair follicles were excluded due to strong autofluorescence that could interfere with quantitative measurements.

### 2.6. Quantitative Real-Time PCR (qPCR) Analysis of Immunomarkers

Quantitative real-time polymerase chain reaction (qPCR) was used to assess the expression of selected immunomarkers. Total RNA was isolated from frozen skin tissue using the RNeasy Mini Kit (Qiagen N.V., Hilden, Germany) according to the manufacturer’s instructions. Tissue samples were pulverized in liquid nitrogen and lysed in RLT buffer containing β-mercaptoethanol (β-ME). When required, further homogenization was performed using the TissueLyser LT (Qiagen N.V., Hilden, Germany). Following centrifugation, the supernatant was mixed with ethanol and applied to spin columns. After the washing and drying steps, RNA was eluted and quantified at 260 nm using a NanoDrop 2000c spectrophotometer (Thermo Scientific™, Waltham, MA, USA).

Complementary DNA (cDNA) was synthesized using the High-Capacity cDNA Reverse Transcription Kit (Applied Biosystems™, Thermo Fisher Scientific, Waltham, MA, USA) according to the manufacturer’s protocol. RNA samples were diluted to match the lowest concentration across all samples. A master mix containing reverse transcription buffer, dNTPs, random primers, reverse transcriptase, RNase inhibitor, and nuclease-free water was prepared. RNA and master mix were combined, briefly centrifuged, and incubated for reverse transcription using a FlexCycler2 thermocycler (Analytik Jena, Jena, Germany). Negative controls lacking reverse transcriptase were included to monitor for genomic DNA contamination.

Expression levels of tumour necrosis factor alpha (TNF-α), cluster of differentiation 14 (CD14), and interleukin-6 (IL-6) were quantified using gene-specific primers (Biomol GmbH, Hamburg, Germany). β-Actin served as the endogenous control for normalization. qPCR was performed using SYBR Green detection on a CFX96™ Real-Time PCR System (Bio-Rad Laboratories Inc., Hercules, CA, USA) with 45 amplification cycles (95 °C denaturation, 60 °C annealing/extension). Amplification specificity was confirmed by melting curve analysis. Relative gene expression was calculated using the ΔΔCT method.

### 2.7. Statistical Analysis

Statistical analyses were performed using GraphPad Prism (Version 10.3.1, GraphPad Software LLC, Boston, MA, USA). Data are presented as median with range or interquartile range (IQR), and 95% confidence intervals (95% CI) are reported where appropriate. Immunohistochemical results are expressed as intensity per cell, whereas qPCR results are expressed as fold change, calculated using the ΔΔCT-method.

Data were assessed graphically for normal distribution, which was consistently observed; therefore, parametric statistical tests were applied. For continuously scaled variables, group differences were analyzed using one-way analysis of variance (ANOVA) with Tukey’s post hoc test for multiple comparison adjustment. Comparisons were conducted between treatment groups and the sham control at each individual time point (3 and 7 days). To compare outcomes between 3 and 7 days, a mixed-effects model with Tukey’s post hoc test for multiple comparison adjustment was used. Statistical significance was defined as an alpha level of 0.05 (5%).

## 3. Results

### 3.1. Hematoxylin–Eosin (HE) and Collagen (Movat Pentachrome—MP) Staining

To obtain an overview of sample preparation and the quality of imaged tissue sections, HE staining was performed for all samples and qualitatively evaluated by microscopy. For postoperative day 3, 5 images for the sham group, 8 for the ONRC group, 7 for the ORC group, and 7 for the GELA group were analyzed. For postoperative day 7, 9 images from the sham group, 5 from the ONRC group, 4 from the ORC group, and 8 from the GELA group were analyzed.

**Figure 2:**
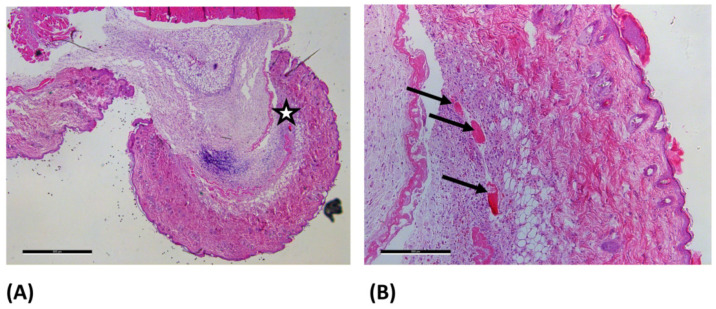
Light microscopy images of the ONRC group at day 7 after hematoxylin-eosin staining. (**A**) 2.5× magnification (scale bar: 500 µm) and (**B**) 10× magnification (scale bar: 500 µm).The enlarged area is marked with a star (☆) in image (**A**). In (**B**) the arrows (→) mark eosinophilic residues, which most likely represent undegraded residues of the used hemostatic agents within the dermis.

In the ONRC and ORC groups, eosinophilic hemostatic residues were detected within the tissue (Figure 2B).

**Figure 3:**
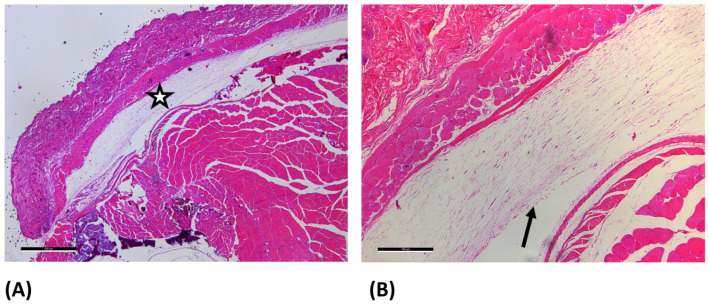
Light microscopy images of GELA group at day 7 after hematoxylin-eosin staining. (**A**) 2.5× magnification (scale bar: 100 µm) and (**B**) 10× magnification (scale bar: 100 µm). The enlarged area is marked with a star (☆) in image (**A**). In figure (**B**) the arrow (→) marks fibrous structure, which most likely represent yet undegraded gelatinous parts of the material embedded during surgery.

In the GELA group, fibrous structures were observed in some samples which were not evident in the other groups (Figure 3B), potentially indicating gelatinous residues of the hemostatic agent. MP collagen staining was additionally performed to support further differentiation; in this staining, extracellular matrix (ECM) components are stained blue.

**Figure 4:**
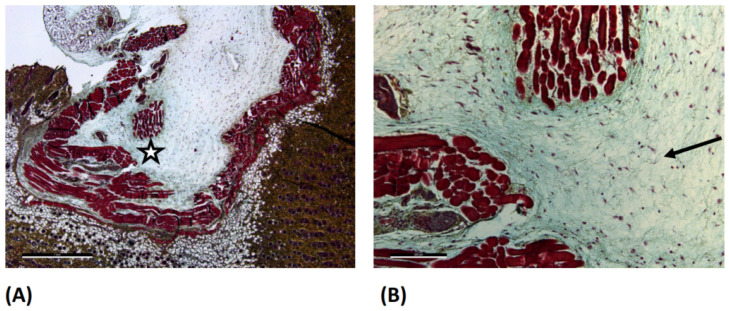
Light microscopy images of GELA group at day 3 after Movat Pentachrome (MP) staining. (**A**) 2.5× magnification (scale bar: 1 mm) and (**B**) 10× magnification (scale bar: 200 µm). The magnified area is marked with a star (☆) in image (**A**). In figure (**B**) the arrow (→) marks the blue stained area, representing increased newly formed extracellular matrix structures (ECM).

The GELA group demonstrated more pronounced ECM staining, suggesting increased collagen deposition relative to the other investigated groups (Figure 4B).

**Figure 5:**
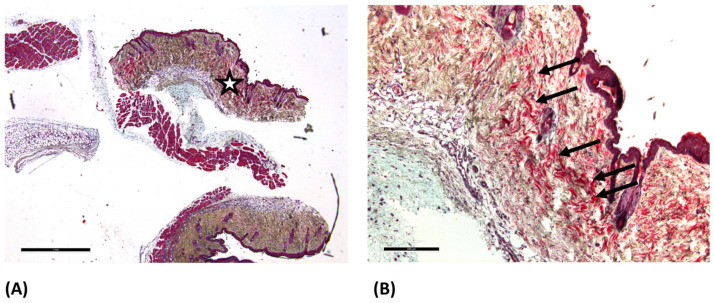
Light microscope images of the OCR group at day 3 after Movat Pentachrome (MP) staining. (**A**) 2.5× magnification (scale bar: 1 mm) and (**B**) 10× magnification (scale bar: 200 µm). The magnified area is marked with a star (☆) in image (**A**). In image (**B**) the arrows (→) marks pronounced streaks of red fibers which potentially represent an increased myofibroblast deposition and activity.

In addition, red-stained fibres were clearly visible within the dermis in all four groups (sham, ORC, ONRC, and GELA; Figure 5B) and were interpreted as most likely representing myofibroblasts within the dermis. However, no distinct increase in deposition or occurrence in any specific group was observed.

### 3.2. Immunohistochemical Evaluation of CD68 and Ki-67

To investigate cell proliferation and macrophage activity as surrogates for regenerative and inflammatory processes, the markers Ki-67 and CD68 were assessed by immunohistochemistry on the postoperative days 3 and 7. As optical differences between dermis and epidermis were apparent during microscopy, these skin layers were analyzed separately.

Significant differences were observed for CD68. These differences may be influenced by biomaterial-specific properties such as degradation behaviour, tissue interaction, and the timing of macrophage recruitment during early wound healing. When comparing dermal intensity between ONRC and ORC on postoperative day 3, ORC demonstrated a significantly increased intensity compared with ONRC (*p* = 0.0489; Δ = 0.019, 95% CI [0.0007; 0.037]). Although both ORC (Δ = 0.014, 95% CI [−0.003; 0.032]) and GELA (Δ = 0.009, 95% CI [−0.009; 0.028]) demonstrated higher intensity than the sham group, these differences were not statistically significant. After 7 days, no significant differences between the investigated materials were detected in the dermis.

**Figure 6:**
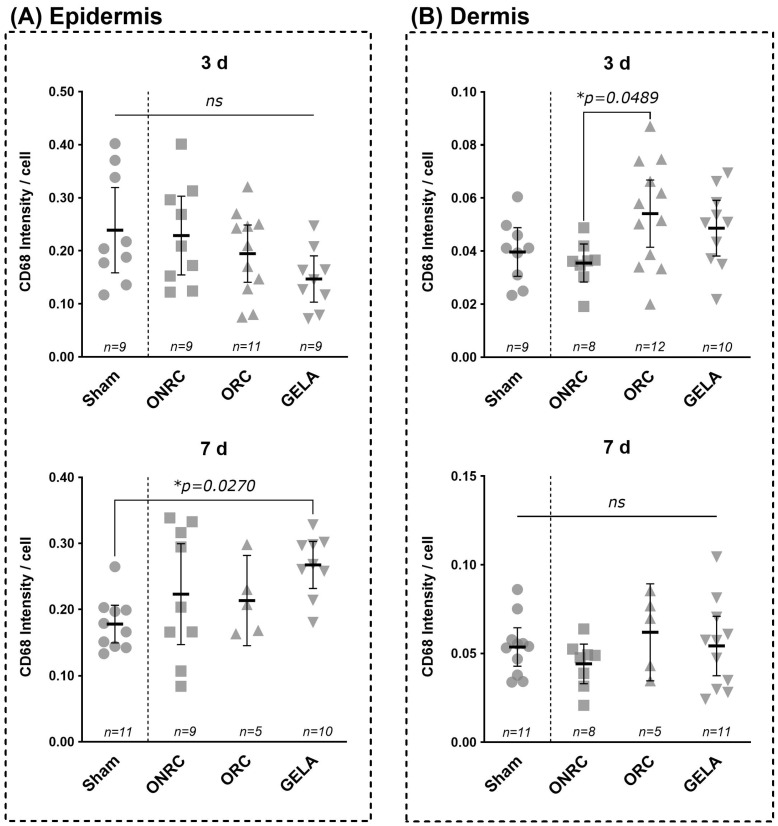
Comparison of CD68 intensity between groups at days 3 and 7 post-surgery. Results for CD68 intensity after staining are shown for epidermis (**A**) and dermis (**B**) for the time points of the 3rd (**upper row**) and 7th (**lower row**) postoperative day. While all intervention groups showed a lower mean CD68 intensity compared to the sham control, no significant difference was observed in the epidermis at the 3rd postoperative day (**A**). After 7 days results were inverted with GELA showing a significantly higher CD68 intensity compared to the sham group (Δ = 0.089, 95% CI [0.008; 0.171], *p* = 0.0270). In the dermis (**B**) a significant difference was observed between ONRC and ORC, with higher intensity in ORC (Δ = 0.019, 95% CI [0.0007; 0.037], *p* = 0.0489) after 3 days. At the 7th postoperative day, no significant difference was observed between the investigated groups (ns—not significant, * *p* < 0.05).

In the epidermis, all tested materials demonstrated lower CD68 intensity than the sham group; however, these differences were not statistically significant. The absence of statistical significance suggests that these differences are unlikely to reflect biologically relevant suppression of macrophage activity, but rather normal spatial variability in epidermal immune cell distribution. On postoperative day 7, the GELA group (0.267 ± 0.046) demonstrated a significantly higher intensity than the sham group (0.178 ± 0.040; Δ = 0.089, 95% CI [0.008; 0.171]; *p* = 0.027; Figure 6).

**Figure 7:**
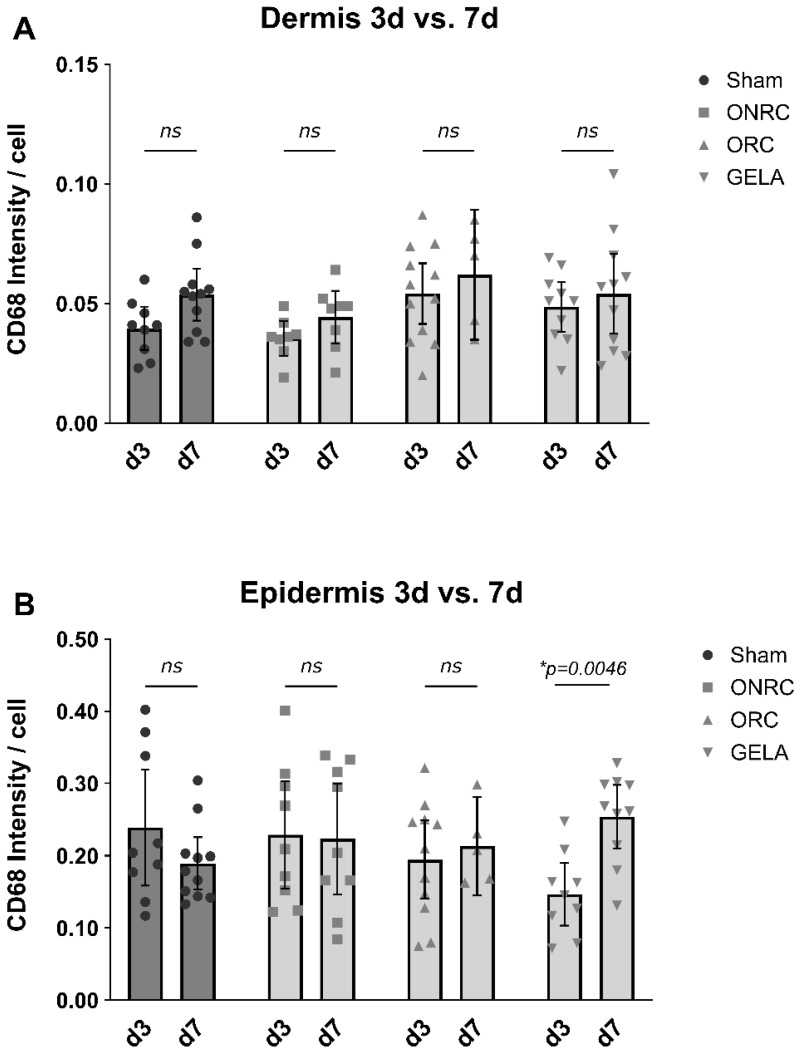
Comparison of difference in CD68 intensity between the 3rd and 7th postoperative day. CD68 intensity after staining are shown for dermis (**A**) and epidermis (**B**) compared between the 3rd and 7th postoperative day for each tested material and the control (sham). While no significant difference between days 3 and 7 were observed in the dermis regarding CD68 intensity, a significantly higher CD 68 intensity was observed in the epidermis after 7 days as compared to after 3 days in the GELA group ((**B**); Δ = 0.107, 95% CI [0.0342; 0.179], 0. *p* = 0.0046) (ns—not significant, * *p* < 0.05).

Comparing epidermal intensity between postoperative days 3 and 7 for each material showed a significant increase within the GELA group, with CD68 expression significantly elevated on day 7 compared with day 3 (Δ = 0.107, 95% CI [0.034; 0.179]; *p* = 0.0046; Figure 7). Although CD68 intensity was generally higher on day 7 than on day 3 in the dermis, this change was not statistically significant. Collectively, these findings suggest biomaterial-dependent modulation of macrophage presence during early wound healing rather than excessive inflammatory activation and are consistent with previous studies demonstrating that biomaterial composition and degradation behavior critically influence macrophage recruitment and proliferative responses during early wound healing [7,19,20].

**Figure 8:**
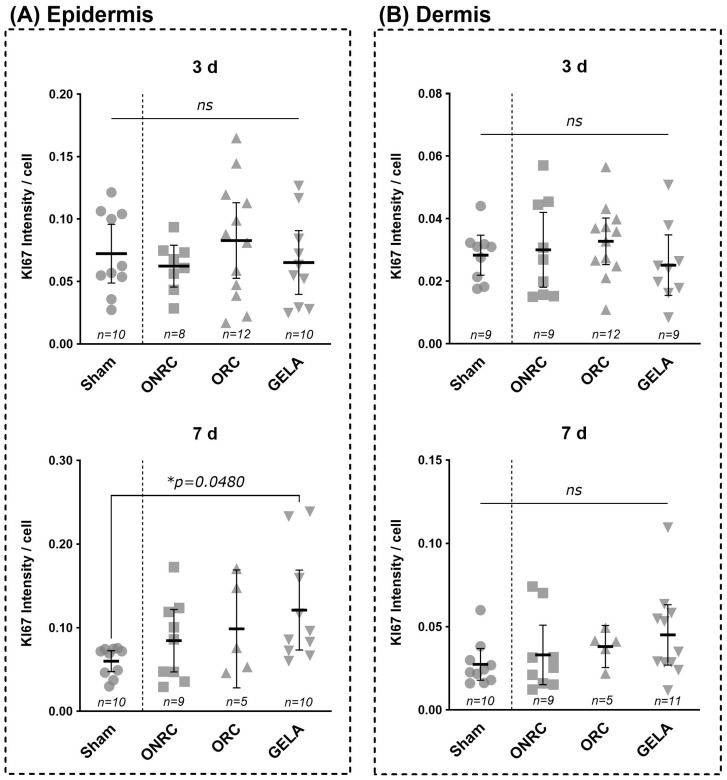
Comparison of KI67 intensity between groups at days 3 and 7 post-surgery. Results for KI67 intensity after staining are shown for epidermis (**A**) and dermis (**B**) for the time points of the 3rd (**upper row**) and 7th (**lower row**) postoperative day. No significant differences were observed between the groups investigated at the 3rd postoperative day, neither in the Epidermis (**A**) nor the dermis (**B**). After 7 days all intervention groups showed higher levels of KI67 intensity compared to sham, both in the epidermis and the dermis, however only GELA showed significantly elevated levels (Δ = 0.061, 95% CI [0.0004; 0.122], *p* = 0.0480) in the epidermis (ns—not significant, * *p* < 0.05).

For Ki-67 intensity, significant differences were detected only in the epidermis on postoperative day 7 when comparing the GELA group (0.121 ± 0.067) with the sham group (0.060 ± 0.018). Ki-67 expression was significantly higher in the GELA group (Δ = 0.061, 95% CI [0.0004; 0.122]; *p* = 0.048; Figure 8). The restriction of significant Ki-67 differences to the epidermis at day 7 is consistent with the temporal progression from inflammation towards epidermal regeneration and proliferative repair. No significant differences were observed within the dermis at either investigated time point.

**Figure 9:**
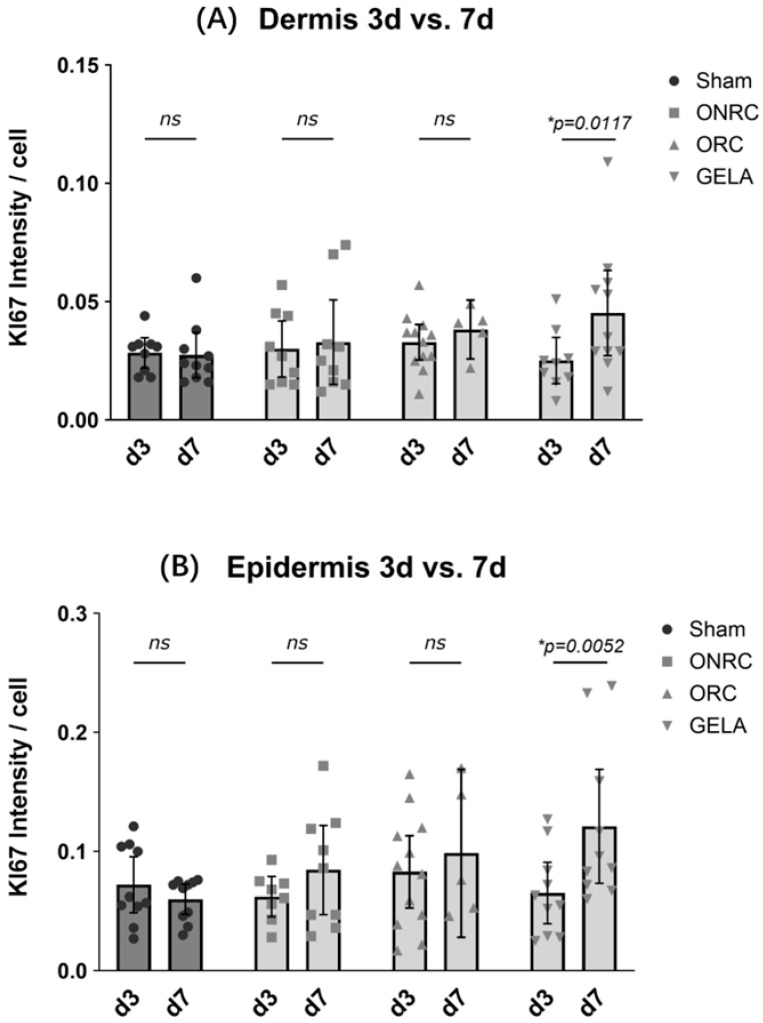
Comparison of difference in KI67 intensity between the 3rd and 7th postoperative day. Results for KI67 intensity after immunohistochemistry staining are shown for dermis (**A**) and epidermis (**B**) compared between the 3rd and 7th postoperative day for each tested material and the control (sham). A significant difference between days 3 and 7 was observed for the GELA group with a higher KI67 intensity at the 7th postoperative day both in der dermis ((**A**); Δ = 0.020, 95% CI [0.005; 0.035], *p* = 0.0117) and the epidermis ((**B**); Δ = 0.056, 95% CI [0.017; 0.095], *p* = 0.0052) (ns—not significant, * *p* < 0.05).

When comparing Ki-67 intensity between postoperative days 3 and 7, GELA demonstrated significantly elevated expression at day 7 in both the dermis (Δ = 0.020, 95% CI [0.005; 0.035], *p* = 0.0117) and the epidermis (B; Δ = 0.056, 95% CI [0.017; 0.095], *p* = 0.0052; Figure 9). While the other tested materials also demonstrated increased Ki-67 levels on day 7 compared with day 3, and the sham group showed lower levels on day 7 compared with day 3, these differences were not statistically significant.

To improve clarity and facilitate between-group comparisons, key quantitative immunohistochemical findings are summarized in (Table 2).

### 3.3. Quantification of Inflammatory Markers IL-6, TNF-α, and CD14

To quantify differences in inflammatory markers between the four investigated groups, real-time qPCR was performed for IL-6, TNF-α, and CD14. To assess changes in expression over time, fold-change values were compared between postoperative days 3 and 7.

**Figure 10:**
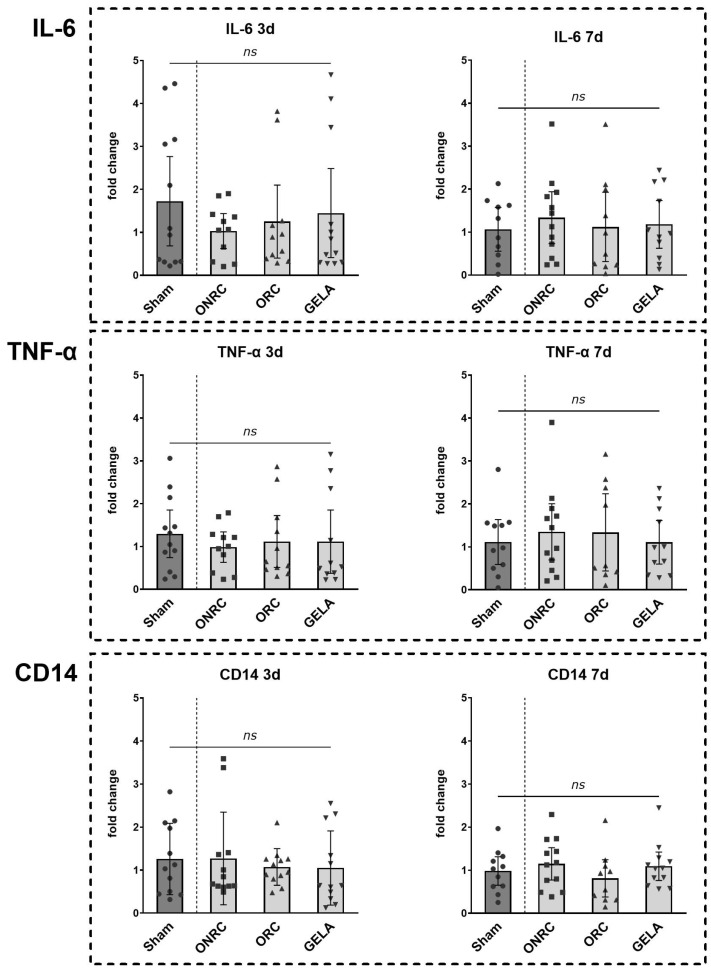
Comparative evaluation of inflammatory marker levels between tested materials. IL-6, TNF-α and CD14 were evaluated at the 3rd and 7th postoperative day using qPCR and measured levels for each intervention group compared. No statistically significant difference was observed for either marker between the different intervention groups at 3 or 7 days postoperative (ns—not significant).

No significant differences were detected between the biomaterial groups (ONRC, ORC, and GELA) or compared with the sham group (Figure 10). This may be explained by the early and transient expression profiles of IL-6 and CD14, which often peak within the first hours after injury and may have returned towards baseline by days 3 and 7 [5,21]. In contrast, TNF-α may exhibit a broader temporal expression window during early wound healing, potentially accounting for the modest but non-significant elevation observed [5,22].

**Figure 11:**
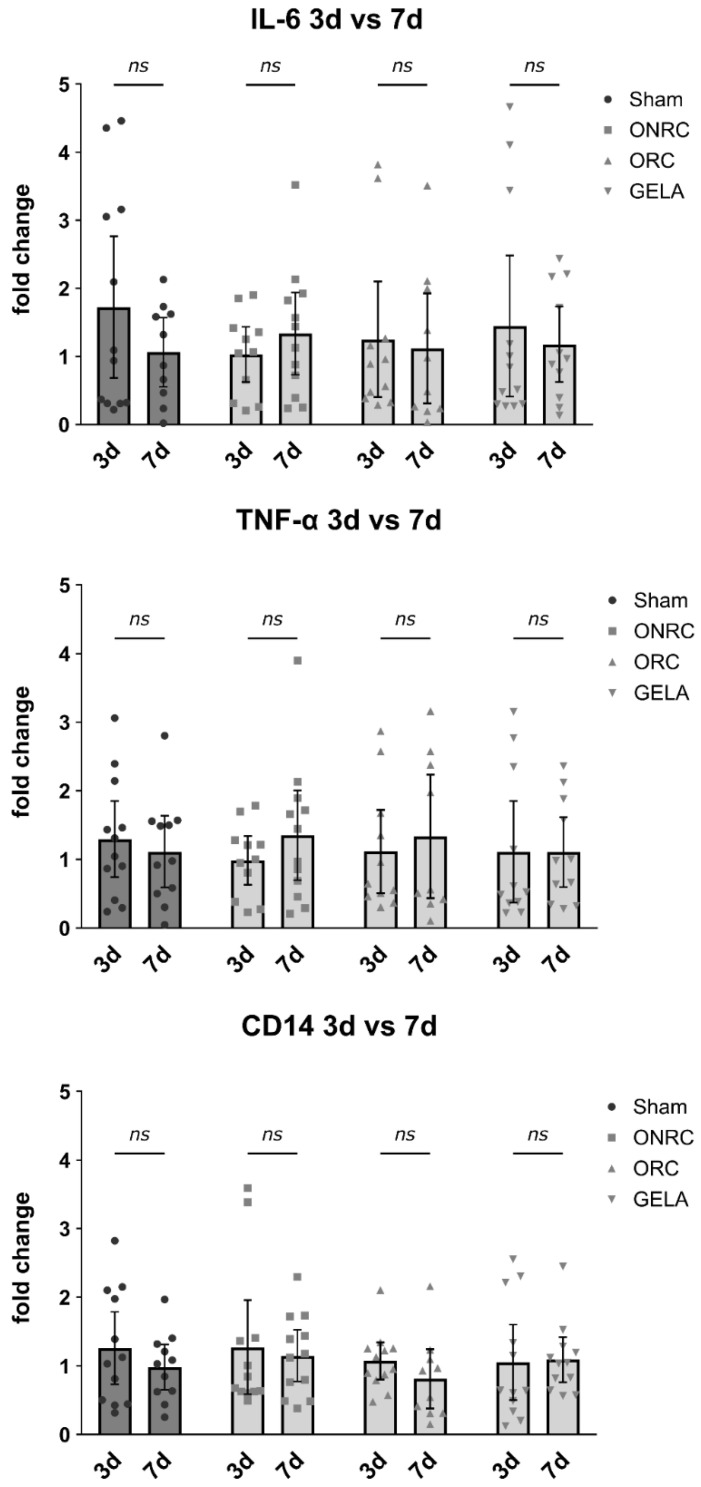
Comparison of difference in inflammatory marker levels between the 3rd and 7th postoperative day. Results for IL-6, TNF-α and CD14 levels measured via qPCR compared between the 3rd and 7th postoperative day for each tested material and the control (sham). No statistically significant difference was observed for either marker between days 3 and 7 for the investigated groups (ns—not significant).

Results for IL-6 are shown for postoperative day 3 (sham: 1.724 ± 1.637; ONRC: 1.030 ± 0.607; ORC: 1.250 ± 1.266; GELA: 1.448 ± 1.630) and postoperative day 7 (sham: 1.064 ± 0.711; ONRC: 1.335 ± 0.951; ORC: 1.120 ± 1.130; GELA: 1.179 ± 0.826). Overall, IL-6 levels were slightly higher on day 7 than on day 3 relative to the sham group (Figure 11).

For TNF-α, a similar pattern was observed, with slightly higher levels compared to sham on day 7. On postoperative day 3, levels were 1.296 ± 0.874 for the sham group, 0.985 ± 0.529 for ONRC, 1.115 ± 0.902 for ORC, and 1.111 ± 1.100 for GELA, whereas on postoperative day 7, levels were 1.113 ± 0.778 for the sham group, 1.350 ± 1.031 for ONRC, 1.337 ± 1.170 for ORC, and 1.107 ± 0.757 for GELA (Figure 11).

For CD14, no pattern was observed in the results for postoperative day 3 (sham: 1.258 ± 0.828; ONRC: 1.272 ± 1.076; ORC: 1.073 ± 0.426; GELA: 1.050 ± 0.864) or postoperative day 7 (sham: 0.983 ± 0.493; ONRC: 1.149 ± 0.592; ORC: 0.812 ± 0.604; GELA: 1.092 ± 0.519; Figure 11).

These observations are consistent with previous studies demonstrating that pro-inflammatory cytokines such as IL-6 and CD14 are typically characterized by an early and transient expression profile following tissue injury, with peak levels occurring within the first hours after wounding and rapidly declining thereafter. In contrast TNF-α has been reported to exhibit a broader temporal expression window during early wound healing, which may explain the modest but non-significant elevation observed at later time points in the present study. Moreover, the absence of sustained cytokine upregulation at days 3 and 7 is in line with physiological wound healing processes and suggests that none of the investigated biomaterials induced an excessive or prolonged inflammatory response [5,7].

To further facilitate comparison of inflammatory gene expression between biomaterial groups and time points, qPCR results are summarized in (Table 3).

## 4. Discussion

Hemostats are commonly used for intraoperative bleeding control [16,23]. In this study, oxidized regenerated (ORC; Tabotamp^®^) and oxidized non-regenerated (ONRC; Resorba CELL^®^) cellulose-based hemostats were used and compared with a gelatin-based hemostat (GELA; GELITA TUIFT-IT^®^) to assess effects on key components of early-phase wound healing in an in vivo model in mice. Cellulose is manufactured from glucopyranose, which polymerizes via β-glucosidic bonds [24]. In ORC, fibre formation first occurs using dinitrogen tetroxide before oxidation, giving the material its smooth structure, with polyuronic acid as the main component. In contrast, ONRC retains an unorganized and therefore frayed fibre structure [16]. In addition to enzymatic degradation, the fibrous component of these hemostats requires degradation through phagocytosis by macrophages [24,25]. ORC and ONRC directly induce platelet activation and aggregation, thereby promoting clot formation [26].

Gelatin-based hemostatic agents have undergone minimal changes and have been well established across multiple surgical specialties since their introduction in the 1940s [27,28]. Gelatin is absorbable and controls bleeding by tamponade, acting as a ‘hemostatic plug’ [28,29,30]. In clinical settings, recent studies have reported positive effects on wound healing when gelatin is used alone or in various material combinations in wound dressings [31,32,33], potentially mediated by favourable effects on wound-healing cytokines such as VEGF in in vitro studies [34]. In a previous in vitro study by our group, GELA did not negatively affect wound-healing processes with respect to pH and cytokine levels (TGF-β, TNF-α), as well as fibroblast viability and migration, in contrast to cellulose-based hemostats [26].

In an in vitro study, ORC and ONRC demonstrated reduced fibroblast proliferation and cell migration during the first two weeks compared with controls. Moreover, ONRC resulted in lower cell proliferation and migration compared with ORC [35].

A former in vitro study by our group evaluated the effects of ORC, ONRC, and GELA on wound-healing components in vitro and showed reduced cell viability and migration for ORC and ONRC compared with GELA and controls, indicating impaired wound healing associated with cellulose-based hemostats. In that study, GELA showed prolonged structural integrity compared with ORC and ONRC, but no effect on cell metabolic activity [26].

In the present study, hematoxylin–eosin staining revealed that partial remnants of ONRC and ORC were still present in the wound tissue after 3 and 7 days (Figure 2B), whereas this was not observed for GELA (Figure 3B), suggesting that GELA may be more readily degradable during the early phase of wound healing. Conversely, fibrous structures were visible in some cases after GELA, in contrast to ONRC and ORC, which may imply that GELA use could contribute to scarring in early wound healing. Histological comparisons of residual biomaterial, extracellular matrix formation, and fibrous structures were primarily descriptive, as no predefined semi-quantitative scoring system was applied.

As a structural protein, collagen determines the mechanical properties and stability of connective tissue. During hemostasis and the inflammation phase, collagen physically promotes hemostasis, absorbs wound exudate, and supports wound cleansing through inflammation-inducing proteases and cytokines. During the granulation phase, collagen provides the basic scaffold for stable wound closure and serves as a matrix for fibroblast migration and proliferation [36,37].

In our study, blue collagen staining was more pronounced in the GELA group (Figure 4B), suggesting that GELA enhances collagen production during the early (inflammatory) phase of wound healing, likely promoted by fibroblast proliferation, which in this study is supported by increased Ki-67 expression [38,39]. In addition, a study in diabetic mice demonstrated significant beneficial effects of treatment with photo-crosslinked gelatin combined with bFGF on wound closure, including increased fibroblast numbers with consequent collagen production and enhanced granulation tissue formation, compared with bFGF alone [40].

In the context of wound healing, increased collagen production may have beneficial effects by stabilizing the extracellular matrix; however, it may also increase scarring. That said, the mice in this study did not clinically show any tendency towards more pronounced scarring in the GELA group. Furthermore, several studies suggest that gelatin-based materials may reduce scar tissue formation, as shown in myocardial infarction-induced mice [41] and in applications for spinal cord injury [42]. Therefore, the present results indicate at least no negative effect on wound healing during the early phase.

The inflammatory phase of wound healing is mediated by various factors to attract and activate relevant cellular components. TNF-α, a key mediator of the early inflammatory phase, is produced and released by activated macrophages, keratinocytes, and other cells, promoting the attraction and activation of additional immune cells (e.g., neutrophils) at the wound site. These cells clear debris and pathogens, thereby preparing a clean wound bed for subsequent granulation and epithelialization. In addition, TNF-α enhances fibroblast proliferation, supporting granulation tissue formation and extracellular matrix (ECM) contraction [22,38].

A former in vitro study showed reduced TNF-α values in the presence of ORC and ONRC compared with GELA (within a maximum time window of 24 h), proposing reduced fibroblast metabolic activity, which would be required for wound healing [26]. This observation is consistent with a previous in vitro study suggesting inhibition of relevant wound-healing processes by ORC and ONRC, including fibroblast proliferation and migration [35].

As in this study none of the hemostatic biomaterials altered TNF-α release compared with sham during the early postoperative phase (Figure 10), these findings suggest that the hemostats used may not affect the inflammatory phase during early wound healing.

IL-6 is a proinflammatory cytokine that increases after surgery or trauma [43] and plays a pivotal role in (dermal) wound healing [21,44,45]. Its importance has been convincingly demonstrated in IL-6-knock-out mice, which exhibited reduced expression of factors such as IL-1 and VEGF and showed up to three-fold delayed wound healing compared with wild-type mice [21,45]. Moreover, administration of anti-IL-6-antibody led to significantly delayed wound healing in wild-type mice, underscoring the regulatory role of IL-6 in “leukocyte infiltration, angiogenesis and collagen accumulation” [21]. Treatment with recombinant murine IL-6 has also been shown to restore wound healing in immunosuppressed mice [45]. In diabetic mice, altered IL-6 expression, in some settings accompanied by increased IL-6 levels, has been suggested to contribute to delayed diabetic wound healing [44].

In clinical contexts, IL-6 is elevated after surgery or tissue trauma and is associated with injury severity and mortality [46].

In our study, no hemostatic biomaterial resulted in significant changes in IL-6 after days 3 and 7 (Figure 10), suggesting that the biomaterials used do not substantially alter wound-healing phases when left in situ.

A possible explanation is the temporal expression profile of proinflammatory cytokines such as IL-6 and TNF-α, which typically peak within the first hours to one or two days after injury and may therefore not be captured at days 3 and 7. Accordingly, the absence of significant cytokine upregulation may indicate that none of the tested biomaterials induced an excessive or pathological inflammatory response during early wound healing.

CD68 is a surface molecule expressed on macrophages, which play a key role in wound-healing regulation [47,48,49]. As CD68 is a pan-macrophage marker, increased signal does not permit discrimination between inflammatory and reparative macrophage phenotypes.

CD14 is a receptor for bacterial wall products and is expressed on monocytes and macrophages, which are critical cellular mediators of the inflammatory phase of wound healing [50,51,52]. Upon contact with bacterial lipopolysaccharides (LPS), CD14 presents LPS to toll-like receptor 4 (TLR4) on monocytes and macrophages, which induces the release of proinflammatory cytokines (e.g., TNF-Alpha and IL-6) and promotes further recruitment of inflammatory cells to the wound site [47]. The relevance of CD14-carrying immune cells has been demonstrated in burn injury patients, in whom CD14 upregulation correlated with severity outcomes and hypertrophic scar development [50].

While CD14 levels did not change significantly (Figure 10 and Figure 11), ORC significantly increased CD68 levels in the dermis after day 3 compared with ONRC (Figure 6). CD68 expression alone does not indicate an exclusively inflammatory response, as it does not distinguish between classically activated (M1) and alternatively activated (M2) macrophages.

Therefore, the increased CD68 signal observed, particularly in the GELA group at day 7, may also reflect macrophage involvement in tissue remodelling and reparative processes rather than sustained inflammation. As seven days falls within the proliferative phase of wound healing, this pattern is consistent with M2 macrophage recruitment, which may support tissue regeneration [3,9,53,54]. This interpretation is aligned with the above-mentioned Ki-67 findings and the suggested increase in fibroblast proliferation after seven days in GELA-treated mice.

Enhanced CD68 expression has been reported in an in vivo rat skin wound model, in which a significant increase in M2 macrophages was observed after 8 days of treatment with a ‘physicochemical double-cross-linked gelatin hydrogel’ compared with control wound dressings, supporting the biological plausibility of our findings [55].

In an in vivo study of rats with spinal cord injury, local implantation of neural stem cell derived extracellular matrix-modified gelatin methacryloyl (ECM/GelMa) resulted in beneficial anti-inflammatory effects, including significantly lower proinflammatory M1 expression and higher anti-inflammatory M2 expression 4 weeks after implantation [56]. A prior study by this group reported reduced glial scarring after GelMa application in spinal cord regeneration [42]. Additional beneficial effects of gelatin materials have been shown in a mouse model after induced myocardial infarction, in which GelMa treatment led to reduced scar tissue formation and lower CD68 expression compared with untreated mice, suggesting inflammation reduction [41].

It is well established that wound-healing phases overlap, with the inflammatory phase progressively transitioning into the tissue formation phase, during which cellular proliferation is initiated and sustained [4].

KI-67 is a proliferation marker involved in cell-cycle regulation and mitosis [57]. It has a short half-life (maximum 1.5 h) and is therefore detected in ‘active phases of the cell cycle’, particularly in early mitosis, but not in resting cells [58]. However, Ki-67 is a non-specific proliferation marker and does not identify the proliferating cell type. It has clinical relevance in various tumour types, where it is used to estimate cancer cell proliferation and associated prognosis [58,59]. In the context of wound healing in mice, Ki-67 has been used as an indicator of fibroblast proliferation in hypertrophic scar tissue using immunofluorescence [60].

Comparable effects on fibroblast and keratinocyte proliferation in response to gelatin-based biomaterials have been reported in previous in vitro and in vivo studies, supporting the biological plausibility of the present findings [39,40,61,62]. In our study, GELA induced a significant increase in Ki-67 in the epidermis after 7 days compared with the sham group (Figure 8). In addition, Ki-67 levels increased significantly after 7 days in both dermis and epidermis compared with 3 days, whereas ONRC and ORC did not change Ki-67 levels (Figure 9). Based on the anatomical localization (epidermis and dermis) and the timing at day 7, the increased Ki-67 signal in the GELA group likely reflects enhanced proliferation of resident skin cells such as fibroblasts and/or keratinocytes. This supports the interpretation that GELA positively influences cell proliferation, consistent with a previous report of significantly increased fibroblast proliferation using an alginate–gelatin cross-linked hydrogel compared with alginate hydrogel without gelatin in in vitro cell–material interaction tests [61]. In an in vitro 3D skin model, a gelatin hydrogel with moderate gelatin concentrations (5% and 8%) was suggested as a favourable carrier material for human dermal fibroblast ‘adhesion, viability and migration’ [62]. However, a previous in vitro study by our group showed diminished fibroblast proliferation 7 and 14 days after application of gelatin-based hemostats [39]. An in vivo study in diabetic mice reported comparable wound-healing potential for Genocel (gelatin hydrogel nonwoven fabrics) used as a skin substitute for ulcers compared with the conventionally used Pelnac skin substitute [53].

In the current study, the indicated influence of GELA on cell proliferation may contribute positively to wound healing, while potentially also increasing scar tissue formation. However, the GELA-treated mice did not clinically show hypertrophic scar formation. This finding is interpreted as indicating enhanced regenerative or reparative activity rather than a hyperplastic or pathological response, as no abnormal tissue architecture or dysplasia was observed.

## 5. Conclusions

This study demonstrated that leaving ORC, ONRC, or GELA in situ did not adversely affect the early phase of wound healing in the investigated in vivo model.

Specifically, CD68 expression was significantly increased in the ORC group at day 3 and in the GELA group at day 7 compared with sham controls, while Ki-67 expression in the epidermis was significantly elevated in the GELA group at day 7 (0.121 ± 0.067 vs. 0.060 ± 0.018 in sham *p* < 0.05). Together, these quantitative findings support the conclusion that ORC and GELA promote reparative cellular responses without impairing early wound healing.

Together, these quantitative findings support the conclusion that ORC and GELA promote reparative cellular responses without impairing early wound healing.

In addition, Ki-67 expression in the epidermis was significantly higher in the GELA group at day 7 compared with sham (0.121 ± 0.067 vs. 0.060 ± 0.018 *p* < 0.05), reflecting enhanced cellular proliferative activity. Although increased proliferation may theoretically contribute to scar formation, no histological or macroscopic evidence of excessive fibrosis or abnormal tissue architecture was observed in the present study.

To comprehensively assess the long-term effects and overall impact of these hemostatic biomaterials, additional in vivo studies focusing on later phases of wound healing are warranted.

## 6. Limitations

Several limitations of this study should be acknowledged. First, CD68 was used as a general macrophage marker and does not allow discrimination between pro-inflammatory (M1) and reparative (M2) macrophage phenotypes. Consequently, increased CD68 expression cannot be interpreted as reflecting inflammation alone, particularly at later time points when reparative processes may predominate. Second, Ki-67 is a non-specific proliferation marker and does not permit identification of the proliferating cell types; therefore, conclusions regarding the specific cellular contributors to the observed proliferative response remain inferential. Third, histological assessments of residual biomaterial, extracellular matrix deposition, and fibrous structures were primarily descriptive in nature, as no predefined semi-quantitative scoring system or digital morphometric analysis was applied. Fourth, gene expression analyses were performed on whole wound tissue, which may have masked localized changes at the biomaterial–tissue interface, and early transient cytokine peaks may not have been captured at the selected sampling time points. Finally, the present study focused on early wound healing and did not assess long-term outcomes such as scar maturation or functional tissue remodelling. Future studies incorporating macrophage subtype-specific markers, cell type–specific proliferation analyses, quantitative histological scoring, earlier sampling time points, and extended follow-up periods are warranted to further elucidate the long-term biological effects of these hemostatic biomaterials.

## Figures and Tables

**Table 1:** Investigated hemostatic biomaterials.

Agent	Abbreviation	Product Name	Manufacturer
Oxidized non-regenerated cellulose	ONRC	RESORBA^®^ CELL	Resorba Medical GmbH, Nürnberg, Germany
Oxidized regenerated cellulose	ORC	TABOTAMP^®^	Ethicon, Johnson & Johnson Medical GmbH, Raritan, NJ, USA
porcine gelatin-based material	GELA	GELITA TUFT-IT^®^	GELITA medical GmbH, Eberbach, Germany

**Table 2:** Immunohistochemical analysis of CD68 and KI-67 fluorescence intensity per cell at postoperative days 3 and 7.

Marker	Tissue Layer	Time Point	Sham	ONRC	ORC	GELA
CD68	Dermis	3 d	n.s.	n.s.	↑ vs. ONRC *	n.s.
CD68	Epidermis	7 d	0.178 ± 0.040	n.s.	n.s.	0.267 ± 0.046 *
KI-67	Dermis	3 d	n.s.	n.s.	n.s.	n.s.
KI-67	Epidermis	7 d	0.060 ± 0.018	n.s.	n.s.	0.121 ± 0.067 *

Quantitative immunohistochemical analysis of CD68 and KI-67 fluorescence intensity per cell in epidermal and dermal layers at postoperative days 3 and 7. Data are presented as mean ± standard deviation. Only statistically significant comparisons are reported with numerical values; non-significant differences are indicated accordingly. (n.s., not statistically significant compared to sham controls. ↑ indicates increased expression. * *p* < 0.05.).

**Table 3:** Quantitative real-time PCR of IL-6, TNF-α, and CD14 in wound tissue at postoperative days 3 and 7.

Gene	Time Point	Sham	ONRC	ORC	GELA
IL-6	3 d	1.724 ± 1.637	1.030 ± 0.607	1.250 ± 1.266	1.448 ± 1.630
IL-6	7 d	1.064 ± 0.711	1.335 ± 0.951	1.120 ± 1.130	1.179 ± 0.826
TNF-α	3 d	1.296 ± 0.874	0.985 ± 0.529	1.115 ± 0.902	1.111 ± 1.100
TNF-α	7 d	1.113 ± 0.778	1.350 ± 1.031	1.337 ± 1.170	1.107 ± 0.757
CD14	3 d	1.258 ± 0.828	1.272 ± 1.076	1.073 ± 0.426	1.050 ± 0.864
CD14	7 d	0.983 ± 0.493	1.149 ± 0.592	0.812 ± 0.604	1.092 ± 0.519

Relative gene expression of IL-6, TNF-α, and CD14 in wound tissue at postoperative days 3 and 7 determined by quantitative real-time PCR. Expression levels were normalized to β-actin and are presented as fold change relative to sham controls. Data are shown as mean ± standard deviation. No statistically significant differences were detected between groups.

## Data Availability

The datasets generated and/or analyzed during the current study are not publicly available because the raw data files contain sensitive experimental information not suitable for public sharing but are available from the corresponding author on reasonable request.

## Abbreviations

α-SMA	Alpha-smooth muscle actin
CD	Cluster of differentiation
DAPI	4′,6-Diamidine-2-phenylindol
ECM	Extracellular matrix
EDTA	Ethylenediaminetetraacetic acid
ELISA	Enzyme-linked immunosorbent assay
FGF	Fibroblast growth factor
GELA	Gelatin-based hemostatic agent (GELITA TUFT-IT^®^)
HE	Hematoxylin-eosin staining
ME	Mercaptoethanol
MP	Movat pentachrome staining
IL	Interleukin
Ki-67	Kiel 67
PFA	Paraformaldehyde
ORC	Oxidized regenerated cellulose
ORNC	Oxidized non-regenerated cellulose
qPCR	Quantitative real-time polymerase chain reaction
ROS	Reactive oxygen species
TGFβ	Transforming growth factor β
TNFα	Tumour necrosis factor α
VEGF	Vascular endothelial growth factor
